# Designed formation of Cu_2_S hierarchical nanostructures as self-supported photoelectrodes for photo-supercapacitors†

**DOI:** 10.1039/d5na00327j

**Published:** 2025-06-27

**Authors:** Ruitong Xu, Muhammad Arif, Guopeng Pan, Lin Xu, Ting Zhu

**Affiliations:** a School of Physics and Electronic Information, Yunnan Normal University 768 Juxian Street Kunming 650500 Yunnan China Xulin13888488199@163.com zhut0002@ynnu.edu.cn; b Yunnan Key Laboratory of Optoelectronic Information Technology, School of Physics and Electronic Information, Yunnan Normal University Kunming 650500 China

## Abstract

Self-charged photo-supercapacitors (PSCs) have garnered significant attention due to their light chargeability, rapid charging capabilities, and compact design. In this study, Cu_2_S photoelectrodes composed of hierarchical nanostructures were synthesised on copper foam (CF) by varying thiourea concentrations (10–120 mg) to modulate the Cu_2_S hierarchical nanostructures and then optimise the photoelectrochemical performance. The results show that the CF@Cu_2_S-100 photoelectrode exhibits the most significant photo-enhanced performance, with areal capacitance increasing under light illumination compared to the dark conditions by 32.98% (from 1140 to 1516 mF cm^−2^) at 10 mA cm^−2^, 50.61% (from 980 to 1476 mF cm^−2^) at 20 mA cm^−2^, and 58.82% (from 918 to 1458 mF cm^−2^) at 30 mA cm^−2^. Over 5000 cycles, the specific capacitance remains stable, with an initial areal capacitance of 550 mF cm^−2^ in the dark and a 22.73% enhancement (675 mF cm^−2^) under illumination with a single wavelength of 365 nm. These findings underscore the significance of thiourea concentration in optimizing the properties of Cu_2_S and enhancing its photo-assisted electrochemical performance for self-charging PSCs.

## Abstract 2

Self-supported Cu_2_S photoelectrodes composed of hierarchical nanostructures were synthesized to promote the electrochemical performances in photo-supercapacitors.

## Graphical abstract

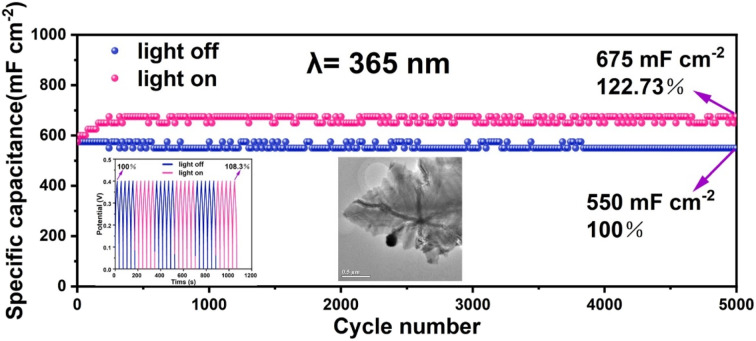

## 1. Introduction

The conversion of solar energy into various forms of energy can help meet global energy needs, as solar energy is an abundant, environmentally friendly, and cost-effective natural resource. While many studies have been conducted on solar radiation, including photovoltaics and photocatalysis, it remains an unreliable but valuable source of energy.^1–3^ The intermittent nature of this energy source, caused by daily and seasonal fluctuations, presents a significant challenge to its continuous supply. The solution to this limitation lies in developing effective strategies for storing and converting solar energy. The use of photo-assisted supercapacitors has emerged as a promising method of storing electrochemical energy by combining solar energy harvesting with electrochemical energy conversion.^4,5^ To achieve high-performance, stable, and efficient PSCs, recent advances are focused on improving charge separation, improving light absorption, and optimizing electrode materials.^6,7^ In recent years, transition metal sulfides (TMSs) have been extensively investigated as high-performance electrode materials due to their low cost, significant interlayer spacing, abundant electrochemically active sites, high theoretical capacities, excellent conductivity, and strong redox reversibility.^8–10^ Following the general reaction, this suggests that transition metals can experience reversible redox reactions in alkaline electrolytes:MS + OH^−^ ⇌ MSOH + e^−^where a transition metal is denoted by M. These redox reactions improve the material's electrochemical performance by promoting ion diffusion and electron transfer, which aid in charge storage. Compared to metal oxides and metal hydro-oxides, metal sulfides have a larger capacitance due to their high electrical conductivity.^11^ In the search for novel materials for energy storage devices, these exceptional qualities of metal sulfides have created new possibilities. Numerous sulfide-based composites have been investigated as photoelectrode materials for photo-supercapacitors, exhibiting notable performance improvements when illuminated.^12–15^ As a photoelectrode for PSCs, cobalt–manganese sulfide has been deposited on TiO_2_ nanotubes to enhance light-driven charge storage. The all-solid-state ASC with a KOH-PVA electrolyte exhibits a capacitance gain of around 70% (42.2 mF cm^−2^) when exposed to light.^12^ With a consistent cycling performance (∼4% capacity loss after 5000 cycles), TMSs meet the need for light-chargeable energy storage systems. Of the several TMSs, copper–sulfur compounds have a steady voltage plateau, a significant theoretical specific capacity, strong electronic conductivity, and good redox reversibility.^16^ Additionally, they have low resistivity, abundant copper reserves, superior low-temperature performance, and a lower electronegativity of sulfur compared to oxygen.^17^ Hydrothermal synthesis of a CuS/SnS_2_@CC photoelectrode resulted in a 17.4% improvement in photo-assisted charge storage. Under illumination, it demonstrated a capacitance of 1155 mF cm^−2^, with 79.81% retention at 3 mA cm^−2^.^18^ The morphology of the synthesised electrode material has a significant impact on its photoabsorption capacity and electrochemical performance. The 3D nanoflower-like MCo_2_O_4_@MCo_2_S_4_@polypyrrole (M = Cu, Mn) core–shell composites feature vertically aligned nanowires and nanosheets grown on a Ni foam coated with a uniform polypyrrole shell.^19^ This hierarchical architecture provides a high surface area and enhanced electroactive interfaces, making it ideal for electrochemical activity. In another study, hierarchical radial Ni_*x*_P nanospheres were uniformly deposited on 3D Ni foam *via* a one-step co-electrochemical method.^20^ This well-defined nanostructure offers abundant active sites and efficient electron transport pathways. For photo-rechargeable capacitors, copper foam-supported Cu_2_S heteroarrays (CS HAs) with sulfur vacancies and leaf-like heterostructures have been created, improving light absorption by forming ternary components.^21^ A 25–35% photo-enhancement was observed in the improved CS HAs, and an areal capacitance of 1440–1048 mF cm^−2^ was measured.

A leaf-like heterostructure of Cu_2_S was grown in this study on a copper (Cu) foam with different thiourea ratios (30 mg, 60 mg, 100 mg, and 120 mg), which regulate the sulfur concentration and the oxidation states of copper (Cu^+^ and Cu^2+^). Cu_2_S primarily exhibits Cu^+^ at lower sulfur concentrations, resulting in decreased stability and conductivity. The partial oxidation of Cu^+^ to Cu^2+^ is promoted by increasing the thiourea ratio, which also improves the electrochemical redox behavior, charge mobility, and electronic structure. Since sulfur balances the oxidation states, it is essential for effective charge separation, improved light absorption, and stability over oxidation–reduction cycles. By facilitating effective charge transfer and ensuring reversible oxidation–reduction reactions, this transition enhances the overall performance, cycling stability, and capacitance of PSCs. To the best of the authors' knowledge, no previous study has reported the use of Cu_2_S as a photoelectrode while systematically investigating the effect of sulfur content introduced *via* varying thiourea concentrations.

## 2. Experimental section

All of the chemicals and solvents in the experiment were of analytical grade and used without further purification.

### 2.1 Preparation of CF@Cu_2_S

Copper foam was cut into 1 × 1 cm^2^ pieces, washed with DI water, dried, and placed in a reaction vessel. The precursor solution was made by dissolving 30 mg, 60 mg, 100 mg, and 120 mg of thiourea in 15 mL of ethanol and 5 mL of DI water while stirring. Each prepared solution was transferred into a reactor containing prepared copper foam, and the sealed reactors were placed in an oven at 180 °C for 16 hours to facilitate the sulfurization process. Following the reaction, the sulfurized CF@Cu_2_S samples were taken out, washed well with DI water to get rid of any remaining precursors, and then left to dry overnight at 60 °C in an oven. The final samples were labeled as CF@Cu_2_S-30 mg, CF@Cu_2_S-60 mg, CF@Cu_2_S-100 mg, and CF@Cu_2_S-120 mg, corresponding to the amount of thiourea used.

### 2.2 Materials characterization

X-ray diffraction (XRD, Rigaku D/max 2500) with Cu Kα radiation (*λ* = 0.154 nm) was used to examine the crystal structures of the formed materials. X-ray photoelectron spectroscopy (XPS, Thermo Scientific K-Alpha ESCALAB 250XI) was used to analyze surface chemical composition and oxidation states using an Al Kα X-ray source (1486 eV). The sample morphologies were examined using scanning electron microscopy (SEM, Quanta FEG 250) with an energy-dispersive spectrometer (EDS) and a transmission electron microscope (TEM, Titan G2 60–300) with elemental mapping capabilities. Monochromatic incident light with a wavelength of *λ* = 365 nm and an alternating voltage of 0.5 V was utilized to conduct the probe.

### 2.3 Electrochemical and photoelectrochemical measurements

Three electrodes were used in an electrochemical workstation (IVIUM, Vertex C. EIS, Ivium Technologies, Eindhoven, The Netherlands) to assess the electrochemical performance of the as-prepared samples. For all electrochemical investigations, a saturated calomel electrode (SCE) served as the reference electrode and a platinum (Pt) plate as the counter electrode in a standard three-electrode arrangement. For all electrochemical measurements, the electrolyte was an aqueous solution of 3 mol KOH. To assess the electrochemical characteristics, galvanostatic charge–discharge (GCD) and cyclic voltammetry (CV) tests were conducted at various scan rates and current densities. The following equation was utilised to determine the areal capacitance (*C*_A_):^22^1
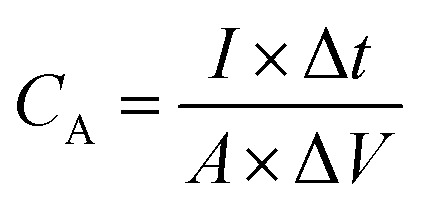
In this case, *C*_A_ stands for areal capacitance (mF cm^−2^), *I* (A) for the discharge current, Δ*t* (s) for the discharge duration, *A* (cm^2^) for the electrode area, and Δ*V* for the discharge voltage window. The photoelectrochemical measurements were conducted using a sealed quartz glass cell with a three-electrode electrochemical setup and the same electrolyte. The light source was a Perfect Light PLS-SXE300 300 W xenon arc lamp featuring a 60 mm spotlight radius and a light power of 50 W. The test cell temperature was kept at 18 °C using cooling water to reduce photothermal effects. Measurements of photo enhancement, such as GCD and CV, were carried out in the presence of chopped light. Linear sweep voltammetry (LSV) was used to detect photocurrent in the range of −0.3 to 0.4 V under chopping light in open-circuit circumstances, using a bias of 0.1 V and a scan rate of 1 mV s^−1^.

## 3. Results and discussion

**Fig. 1:**
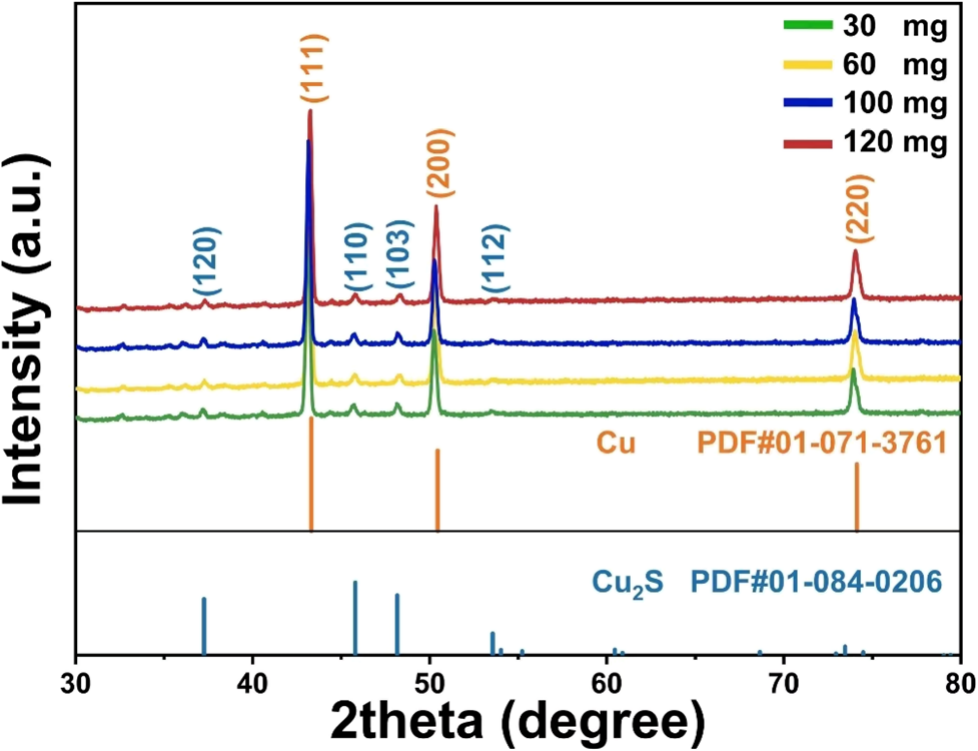
XRD patterns of the CF@Cu_2_S samples.

The XRD analysis confirms the successful synthesis of Cu_2_S on Cu foam, as the diffraction peaks closely match the reference card (PDF# 01-084-0206) (Fig. 1). The materials were successfully synthesized, with varying thiourea concentrations influencing phase formation, crystallinity, and peak shifts, showing a gradual transformation from metallic Cu (PDF# 01-071-3761) to Cu_2_S with increasing sulfur content.

The development of mixed or intermediate copper sulfide phases (such as Cu_2−*x*_S), weak diffraction peaks, and decreased crystallinity are the results of an insufficient sulfur supply for the full sulfurization of the copper substrate at lower concentrations (30–60 mg). Phase-pure Cu_2_S with enhanced crystallinity is formed when the thiourea concentration reaches 100 mg, resulting in a more thorough and consistent sulfurization process, as indicated by sharper and more intense diffraction peaks. This concentration appears to be optimal for minimising structural defects and promoting orderly crystal growth. Although crystallinity remains high at even greater thiourea concentrations (120 mg), excessive sulfur may cause lattice strain or distortions due to over-saturation, which could result in internal tension within the lattice or the deposition of amorphous sulfur. The XRD peak positions are somewhat shifted as a result of these considerations. The long-range order and interatomic spacing are impacted by sulfur-rich or sulfur-deficient (Cu_2−*x*_S) environments, lattice strain, and variations in unit cell characteristics, which are the causes of the observed peak shifts across concentrations.

**Fig. 2:**
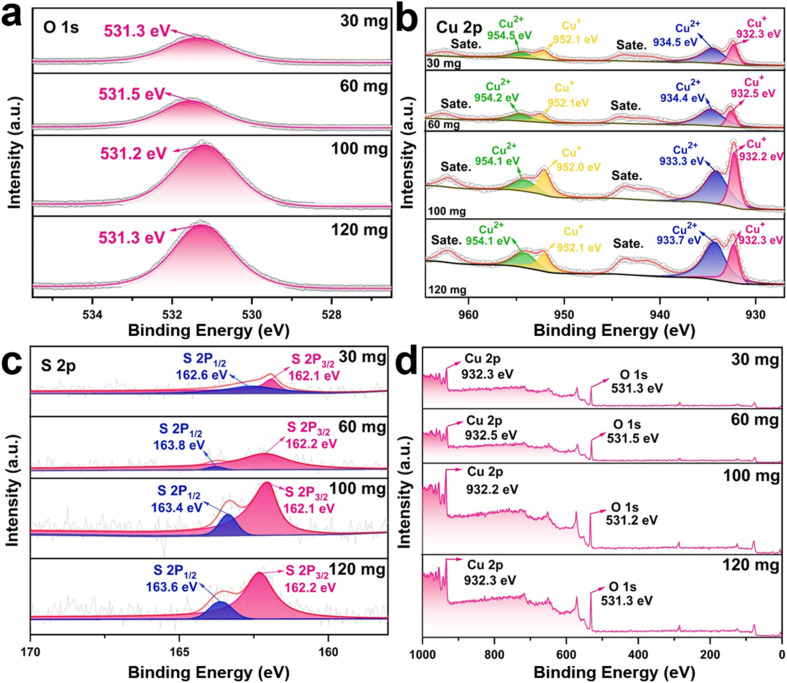
XPS spectra of the CF@Cu_2_S samples: (a) O 1s, (b) Cu 2p, (c) S 2p, and (d) survey spectra.

The XPS spectra show substantial peak shifts in the Cu 2p, S 2p, and O 1s regions as the thiourea concentration increases from 30 mg to 120 mg, showing changes in the chemical states of Cu and S (Fig. 2). The Cu 2p spectra show (Fig. 2b) the Cu 2p_3/2_ peak at 933.7 eV for 30 mg, 933.4 eV for 60 mg, 933.3 eV for 100 mg, and 933.2 eV for 120 mg, indicating a gradual decrease of Cu^2+^ (CuS) to Cu^+^ (Cu_2_S) with increased sulfurization.^23^ A growing percentage of Cu_2_S at higher thiourea concentrations is further confirmed by the Cu^2+^ satellite peaks at 954.6 eV (30 mg), which also slightly shift to 954.1 eV (100 mg) and weaken at 120 mg.^21^ The S 2p_3/2_ peak changes from 162.1 eV (30 mg) to 162.2 eV (60 mg), 162.4 eV (100 mg), and 162.6 eV (120 mg), showing higher Cu–S interactions as well as sulfur incorporation into the lattice (Fig. 2c).^24^ This shift is attributed to an increase in sulfur concentration, which stabilizes the formation of Cu_2_S. In the O 1s spectra, the peak appears at 531.3 eV (30 mg), shifts slightly to 531.5 eV (60 mg), then to 531.2 eV (100 mg), and finally to 531.3 eV (120 mg) (Fig. 2a and d). These modest fluctuations reflect changes in surface oxidation or adsorbed hydroxyl species, which Cu's shifting oxidation states could impact. This shift alters the electronic structure and bonding environment, which is essential to modify the electrochemical properties.

**Fig. 3:**
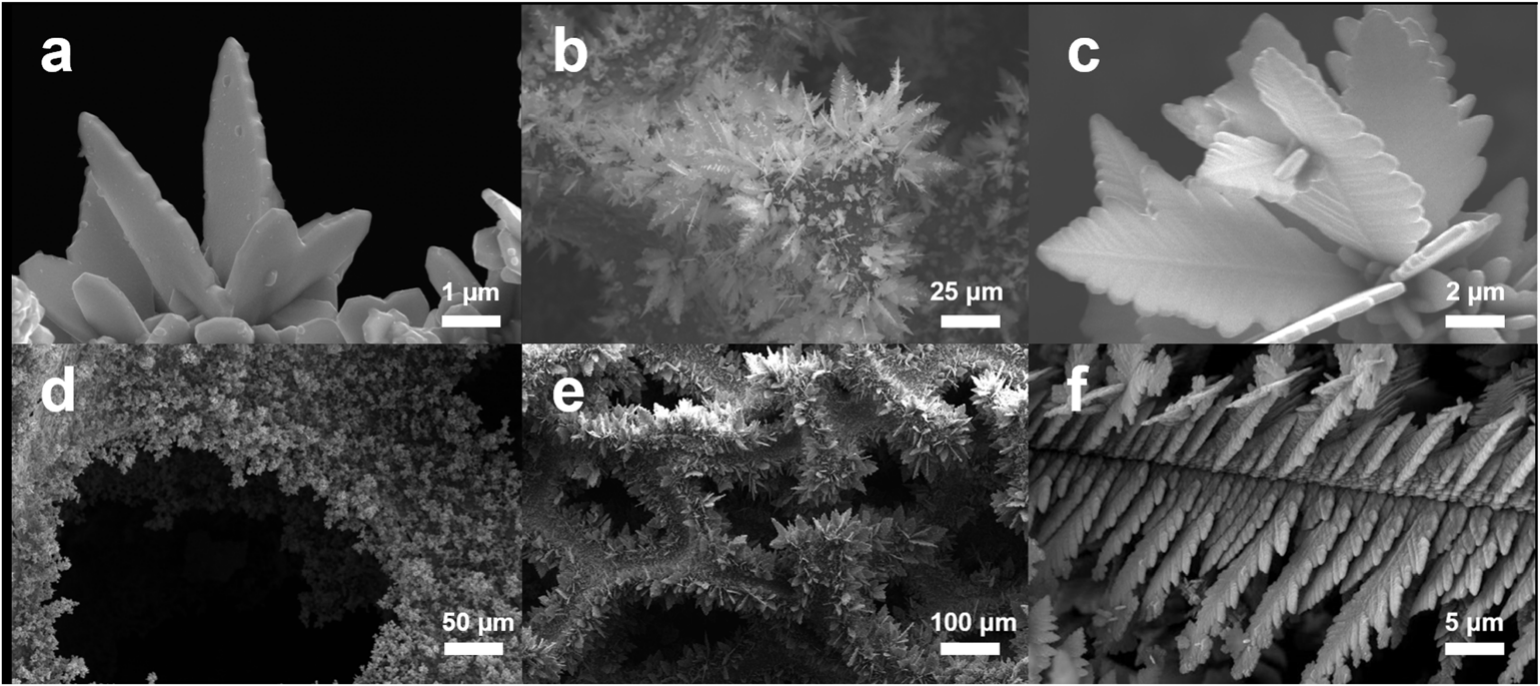
SEM images of the CF@Cu_2_S samples: (a) CF@Cu_2_S-30 mg, (b) CF@Cu_2_S-60 mg, (c and d) CF@Cu_2_S-100 mg, and (e and f) CF@Cu_2_S-120 mg.

The SEM images of CF@Cu_2_S synthesized with different concentrations of thiourea (30, 60, 100, and 120 mg) show notable morphological variations that have a direct impact on the performance of the PSC (Fig. 3). The sparsely dispersed, tiny leaf-like structures seen in CF@Cu_2_S-30 (Fig. 3a) suggest insufficient sulfidation, which could result in inadequate charge transport and restricted light absorption. The network of CF@Cu_2_S-60 (Fig. 3b) is denser and hierarchical, indicating better crystallinity and more electroactive surface area, both of which are advantageous for charge storage and ion diffusion. CF@Cu_2_S-100 (Fig. 3c and d) exhibits distinct, interwoven, fern-like structures with high porosity, which maximize redox reaction sites and light-harvesting capacity, resulting in enhanced electrochemical performance. The CF@Cu_2_S-120 exhibits an overgrown, densely packed morphology with high sulfidation, as shown in Fig. 3e and f. This structure may increase charge recombination and reduce effective ion penetration.

**Fig. 4:**
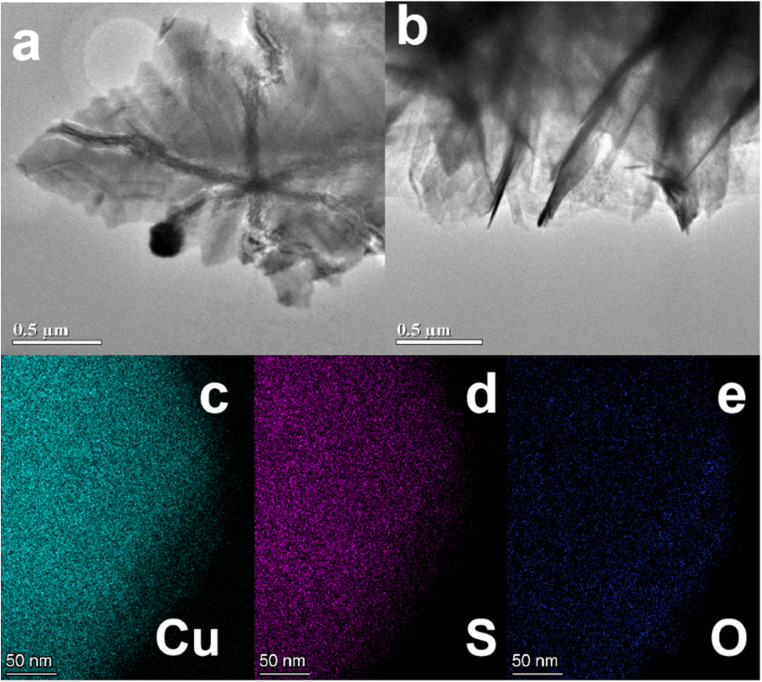
(a and b) TEM images of CF@Cu_2_S-100 sample and (c–e) elemental mappings of Cu, S and O of the sample.

Based on the results obtained from the TEM, we have a deeper understanding of the morphology and structural properties of Cu_2_S at the nanoscale (Fig. 4). The sample appears to have a leaf-like structure with visible folds and wrinkles, which indicates that the structure is layered as shown in Fig. 4a. The image (Fig. 4b) depicts a different perspective, showing sharp edges, stacked regions, and partial stacking of leaves within the heterostructure. As a result of such features, charge transfer pathways are likely to be more efficient and electron mobility is likely to be improved. Moreover, the elemental mapping images (Fig. 4c–e) demonstrate homogeneous distributions of Cu, S, and O throughout the structure, confirming the successful synthesis of Cu_2_S. There is likely to be some partial oxidation at the surface that is responsible for the presence of oxygen.

**Fig. 5:**
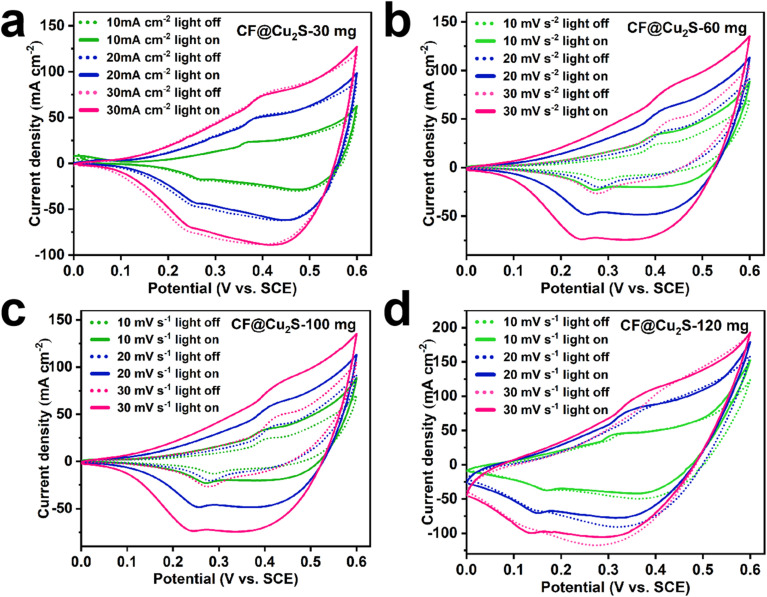
CV curves obtained at different scan rates under dark and light conditions for all the samples: (a) CF@Cu_2_S-30 mg, (b) CF@Cu_2_S-60 mg, (c) CF@Cu_2_S-100 mg, and (d) CF@Cu_2_S-120 mg.

The electrochemical performance of Cu_2_S electrodes produced on Cu foam was evaluated using cyclic voltammetry (CV) under both light and dark conditions. To investigate the effect of sulfur content on electrochemical and photoelectrochemical behaviour, electrodes with varying thiourea concentrations (30 mg, 60 mg, 100 mg, and 120 mg) were prepared. Fig. 5 presents the CV curves recorded within the potential range of 0.0 to 0.6 V (*vs.* SCE) at scan rates ranging from 10 to 30 mV s^−1^. The CV data showed a strong dependency of photoresponsivity and redox activity on sulfur content. The following reactions are principally involved in the redox activity of Cu_2_S in this system:

2 Cu_2_S + 2OH^−^ → 2Cu^+^ + S + H_2_O + 2e^−^

3 Cu^+^ + OH^−^ → CuO + H_2_O + e^−^

4 CuO + H_2_O + e^−^ → Cu^+^ + OH^−^

5 2Cu^+^ + S + 2H_2_O + 2e^−^ → Cu_2_S + 2OH^−^

The absence of an apparent CV response for CF@Cu_2_S-30 under illumination implies that both effective electron transport and the presence of surface-active sites are necessary for this reaction to occur (Fig. 5a). Because of the small contribution of photogenerated charge carriers, CF@Cu_2_S-30 does not play a substantial role in these redox reactions. The low thiourea concentration (30 mg) may lead to insufficient sulfur incorporation, resulting in a less conductive Cu_2_S phase and lower electrochemical activity. A significant increase in the CV area, as shown in Fig. 5b and c, was observed for CF@Cu_2_S-60 and CF@Cu_2_S-100 under illumination, indicating optimized Cu_2_S crystallization. Effective visible-light absorption is made possible by Cu_2_S's low bandgap (1.2–1.5 eV), which encourages the formation of electron–hole pairs.^25^ These photogenerated electrons enhance redox reactions, improving charge separation and reducing recombination, leading to higher capacitance. The improvement in charge separation and reduced recombination in these samples suggest the presence of a well-formed Cu_2_S structure, which supports rapid electron transfer during redox cycling. By enhancing redox processes and lowering recombination, these photogenerated electrons increase capacitance. The presence of a well-formed Cu_2_S structure, which facilitates quick electron transfer during the redox cycle, is suggested by the samples' improved charge separation and decreased recombination. CF@Cu_2_S-120, on the other hand, demonstrated no significant enhancement, most likely as a result of excessive sulfur leading to over-sulfidation, which can introduce recombination centers (Fig. 5d). A surplus of sulfur can cause surface states that trap photogenerated charge carriers and change the electronic structure, which can result in higher recombination, decreased charge mobility, and inadequate band alignment.^26^ The photoactivity of the CF@Cu_2_S-100 sample was verified by the photocurrent response, as shown in Fig. S1,† where the current increased from 0.1 mA cm^−2^ under dark conditions to 1.1 mA cm^−2^ under light conditions.

**Fig. 6:**
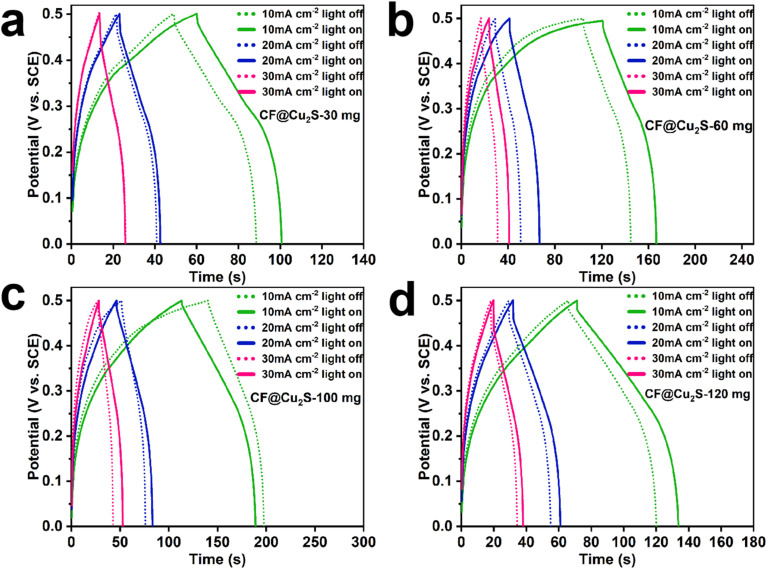
GCD curves obtained at different current densities under dark and light conditions for all the samples: (a) CF@Cu_2_S-30 mg, (b) CF@Cu_2_S-60 mg, (c) CF@Cu_2_S-100 mg, and (d) CF@Cu_2_S-120 mg.

To further investigate the photoelectrochemical behavior and energy storage capabilities of the examined system, GCD analyses were conducted on CF@Cu_2_S-30, CF@Cu_2_S-60, CF@Cu_2_S-100, and CF@Cu_2_S-120 under both dark and light conditions (Fig. 6). The *C*_A_ and their enhancements were calculated using eqn (1), providing a quantitative assessment of the impact of illumination on electrochemical performance (Table 1). The areal capacitance enhancement of CF@Cu_2_S-30 under illumination is negligible at 30 mA cm^−2^ (0%) and very slightly increases at lower currents (3.54% at 10 mA cm^−2^, 1.54% at 20 mA cm^−2^). The limited charge separation and inadequate light-driven energy storage are indicated by the GCD curves' inability to differentiate between light and dark conditions. Moderate photo-enhancement is demonstrated by CF@Cu_2_S-60, which shows capacitance increases of 16.37%, 19.44%, and 12.20% at 10, 20, and 30 mA cm^−2^, respectively. The GCD curves' extended discharge periods under light validate the improved charge storage. A noticeable increase in capacitance under illumination is attributed to the enhanced light absorption and charge transfer facilitated by the comparatively porous and interconnected Cu_2_S network (Table 1). According to this study, CF@Cu_2_S-100 shows the most significant degree of photo-enhancement, with an areal capacitance increase ranging from 1140 to 1516 mF cm^−2^ (32.98%) at 10 mA cm^−2^, 980 to 1476 mF cm^−2^ (50.61%) at mA cm^−2^, and 918 to 1458 mF cm^−2^ (58.82%) at 30 mA cm^−2^. A significant electroactive surface area is provided by the distinct, fern-like nanostructure, which improves charge separation and transport.^27^ The CF@Cu_2_S-100 sample exhibits a remarkably higher calculated capacitance compared to numerous previously reported PSC devices.^12,28–33^

**Table 1:** Areal capacitance enhancement under illumination at different current densities

Samples	Current densities (mA cm^−2^)	Areal capacitance mF cm^−2^ (light off)	Areal capacitance mF cm^−2^ (light on)	Enhancement
CF@Cu_2_S-30	10	792	820	3.54%
	20	780	792	1.54%
	30	774	774	0%
CF@Cu_2_S-60	10	880	1024	16.37%
	20	864	1032	19.44%
	30	820	932	12.20%
CF@Cu_2_S-100	10	1140	1516	32.98%
	20	980	1476	50.61%
	30	918	1458	58.82%
CF@Cu_2_S-120	10	1090	1250	14.68%
	20	1028	1172	14.01%
	30	972	1098	12.96%

The enhanced energy storage performance is confirmed by noticeably longer discharge periods under low-light conditions. When compared to CF@Cu_2_S-100, CF@Cu_2_S-120 shows a decrease in photo-capacitance enhancement, with percentage increases of 14.68%, 14.01%, and 12.96% at 10, 20, and 30 mA cm^−2^, respectively. The GCD curves, which only exhibit modest variations between light and dark situations, support this trend. The GCD data show consistent patterns of charge storage behavior under both dark and light conditions, validating the CV findings. The increased capacitance seen in GCD measurements is consistent with the charge transfer and redox activity aspects found in the CV analysis, demonstrating the validity of the electrochemical performance evaluation. According to these results, the ideal concentration of thiourea for CF@Cu_2_S electrodes is 100 mg. This results in a well-balanced nanostructure that optimizes charge separation, light absorption, and electrochemical performance, leading to maximum capacitance improvement when illuminated. The GCD tests under a switched light on/off mode for the CF@Cu_2_S-100 sample were carried out at 20 mA cm^−2^, where the GCD time can be prolonged under light conditions (Fig. S2†). The charge transfer kinetics of CF@Cu_2_S-30, CF@Cu_2_S-60, CF@Cu_2_S-100, and CF@Cu_2_S-120 samples were examined using Electrochemical Impedance Spectroscopy (EIS). The Nyquist plots (Fig. S3†) show that, in both dark and light circumstances, the charge transfer resistance (*R*_ct_) consistently decreases as the thiourea concentration rises. When no light was present, the *R*_ct_ values for samples containing 30 mg, 60 mg, 100 mg, and 120 mg of thiourea were 1.32 Ω, 1.00 Ω, 0.74 Ω, and 0.45 Ω, respectively. These values further dropped to 1.25 Ω, 0.79 Ω, 0.70 Ω, and 0.45 Ω, respectively, when exposed to light. The gradual decrease in *R*_ct_ suggests that increased thiourea concentration greatly improves electrical conductivity and promotes more effective interfacial charge transfer. Additionally, a drop in *R*_ct_ upon illumination indicates improved mobility and separation of photogenerated charge carriers, which further facilitates the charge transfer process under light-on conditions.

**Fig. 7:**
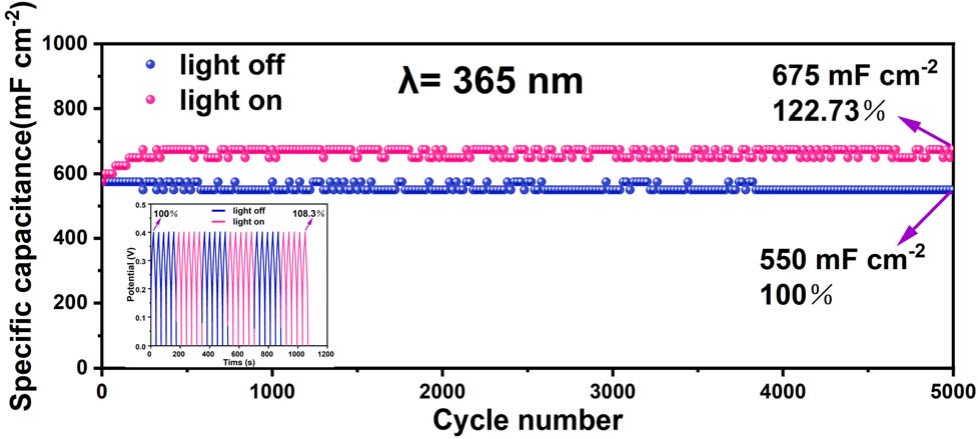
Cycling performances under light off and on conditions of the CF@Cu_2_S-100 sample at a current density of 20 mA cm^−2^ using a light source with a single wavelength of 365 nm.

Next, a considerable photo-induced improvement in charge storage is revealed by the cycling stability analysis of CF@Cu_2_S-100 at a constant current density of 20 mA cm^−2^, exhibiting outstanding long-term performance. Over 5000 cycles, the specific capacitance stays constant, with an initial areal capacitance of 550 mF cm^−2^ in the dark and 675 mF cm^−2^ (a 22.73% enhancement) in the presence of 365 nm illumination, as shown in Fig. 7. The production of photoexcited charge carriers by the Cu_2_S-based electrode material is responsible for this capacitance enhancement.^34^ This increases redox activity and boosts the efficiency of charge storage. Furthermore, the stability over 5000 cycles can be attributed to its well-integrated shape, which reduces structural deterioration and promotes effective charge transport. This photo-enhanced behavior is further supported by the inset GCD curves, which demonstrate a rise in capacitance retention over 1200 seconds. This increase continues in applications where light is turned on and off periodically, with capacitance retention rising from 100% to 108.3%. The response of capacity retention to light irradiation indicates that the electrode materials exhibit high sensitivity and good electrical conductivity.^35^ In summary, the electrochemical activity, phase purity, and crystallinity of Cu_2_S electrodes are all significantly influenced by the concentration of thiourea. At low concentrations, sulfur deficiency inhibits the development of an active Cu_2_S phase, resulting in the absence of photo-enhanced electrochemical activity. A well-structured Cu_2_S network provides stability over a broad range of charge–discharge cycles and facilitates adequate charge storage through light-driven redox reactions at optimal concentrations. On the other hand, sulfur-rich phases that develop at high concentrations lead to increased recombination and lower electrochemical performance. These results underscore the importance of precisely adjusting sulfur precursor concentrations to achieve high-performance Cu_2_S-based photoelectrodes for use in supercapacitors.

Significant performance variations between dark and light conditions are revealed by the cycling stability analysis of the CF@Cu_2_S-100 electrode over 10 000 cycles at a constant current density of 30 mA cm^−2^ (Fig. S4†). After 10 000 cycles in the dark, the electrode's specific capacitance decreases from its initial value of 900 mF cm^−2^ to 675 mF cm^−2^, corresponding to a capacitance retention of 75%. Under light illumination, the initial capacitance increases significantly to 1200 mF cm^−2^ and, after 10 000 cycles, decreases to 975 mF cm^−2^, resulting in a capacitance retention of 81.25%. The enhanced performance of Cu_2_S under illumination is attributed to photo-induced charge carriers, which increase redox activity and decrease charge recombination. Additionally, the conductive Cu foam (CF) network maintains structural integrity and effective electron transport. These findings demonstrate that light activation has a beneficial effect on the electrochemical stability and capacitance retention of the CF@Cu_2_S-100 electrode, indicating that it is a viable option for photo-enhanced energy storage applications. In addition, the CV and GCCD performances of as-prepared materials in a two-electrode system were evaluated under light on and off conditions, respectively, and the results are displayed in Fig. S5.† A larger CV shape and a longer GCCD time can be observed from the results, which reveal the photo-enhanced charge storage property of the as-prepared materials. The photo-conversion efficient was calculated as 0.0026% from the GCCD curve shown in Fig. S5b.†

## 4. Conclusion

In conclusion, a leaf-like Cu_2_S heterostructure was successfully synthesized *via* a hydrothermal method using different thiourea concentrations to optimize its performance as a bifunctional electrode material for photo-supercapacitors. The capacitance enhancement under illumination varies significantly with thiourea concentration. CF@Cu_2_S-30 exhibits negligible photo-enhancement, while CF@Cu_2_S-60 shows moderate increases of 16.37%, 19.44%, and 12.20% at 10, 20, and 30 mA cm^−2^, respectively. The optimal performance is achieved with CF@Cu_2_S-100, demonstrating the highest photo-enhancement, with an areal capacitance increasing from 1140 to 1516 mF cm^−2^ (32.98%) at 10 mA cm^−2^, 980 to 1476 mF cm^−2^ (50.61%) at 20 mA cm^−2^, and 918 to 1458 mF cm^−2^ (58.82%) at 30 mA cm^−2^. However, at higher thiourea concentrations (CF@Cu_2_S-120), excessive sulfur incorporation leads to increased charge recombination, resulting in capacitance enhancements of 14.68%, 14.01%, and 12.96% at 10, 20, and 30 mA cm^−2^, respectively. Cycling stability analysis of CF@Cu_2_S-100 at 20 mA cm^−2^ confirms excellent long-term performance, with capacitance remaining stable over 5000 cycles, increasing from 550 mF cm^−2^ in the dark to 675 mF cm^−2^ (22.73% enhancement) under 365 nm illumination. The well-structured Cu_2_S network at optimal thiourea concentrations ensures stability and efficient charge storage through light-driven redox reactions. In contrast, sulfur-rich phases at higher concentrations promote recombination, lowering electrochemical performance. This study offers valuable insights into optimising sulfur incorporation for enhanced photo-assisted supercapacitors, thereby paving the way for improved light-driven energy storage systems.

## Author contributions

R. T. Xu carried out the experiments, collected, and analyzed the data, M. Arif assisted the data collection and analysis, and wrote the manuscript, G. P. Pan assisted the experiments and data collection, T. Zhu conceived the research idea, supervised the project, analyzed the data and revised the manuscript, L. Xu supervised the project and provided important discussions.

## Conflicts of interest

The authors declare no conflict of interest.

## Supplementary Material

NA-007-D5NA00327J-s001

## Data Availability

The data supporting this article have been included as part of the ESI.†
