# Supplementary material for: Designed formation of Cu2S hierarchical nanostructures as self-supported photoelectrodes for photo-supercapacitors
Source: Nanoscale Adv. 2025 Jun 27;7(16):5058–66. doi: 10.1039/d5na00327j (PMC12246836; doi:10.1039/d5na00327j)
Supplement: NA-007-D5NA00327J-s001 [file NA-007-D5NA00327J-s001.pdf]

Supplementary Information (SI) for Nanoscale Advances.
This journal is © The Royal Society of Chemistry 2025

Supplementary Information

Designed formation of Cu_2S hierarchical nanostructures as self-supported photoelectrodes for photo-supercapacitors

Ruitong Xu, [a†] Muhammad Arif, [a†] Guopeng Pan, [a] Lin Xu [ab*] and Ting Zhu [ab*]

[a] School of Physics and Electronic Information, Yunnan Normal University, 768 Juxian Street, Kunming 650500, Yunnan, China.

[b] Yunnan Key Laboratory of Optoelectronic Information Technology, School of Physics and Electronic Information, Yunnan Normal University, Kunming 650500, China.

*Email: Xulin13888488199@163.com (L. Xu); zhut0002@ynnu.edu.cn (T. Zhu)

[†]These authors contributed equally.

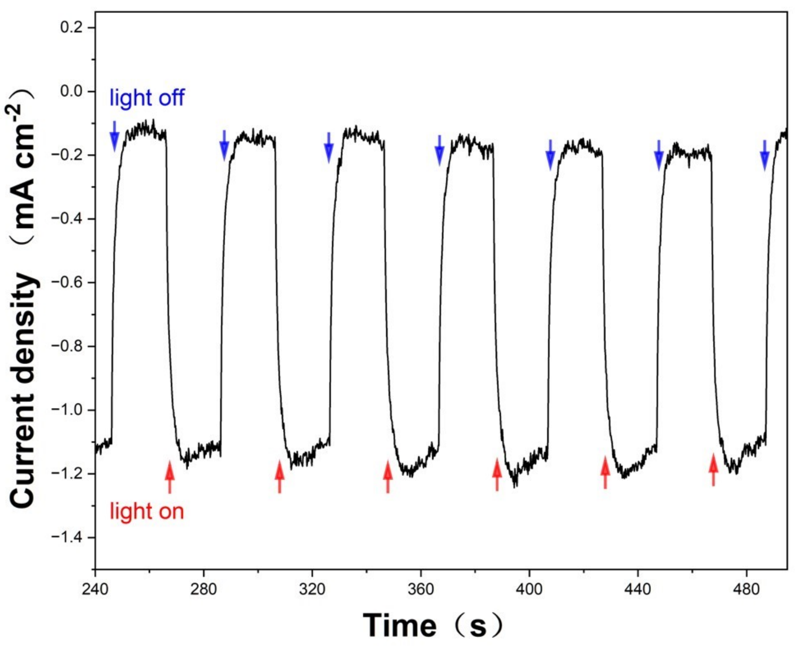

Fig. S1. The photocurrent response of the CF@Cu_2S-100 sample was obtained with a time interval of 20 s.

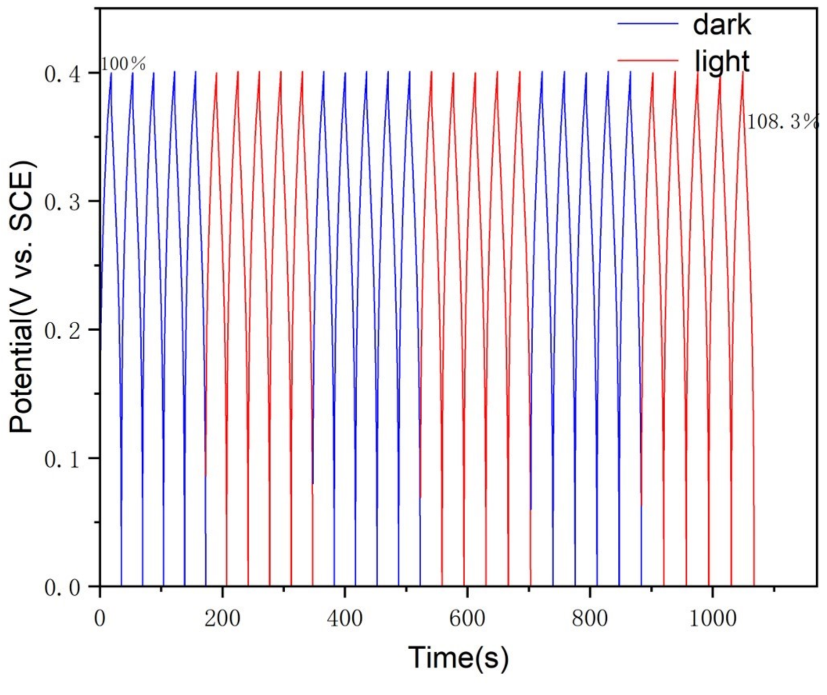

Fig. S2. The GCD curves were obtained at 20 mA cm^{-2} under a switched light on/off mode for the CF@Cu_2S-100 sample.

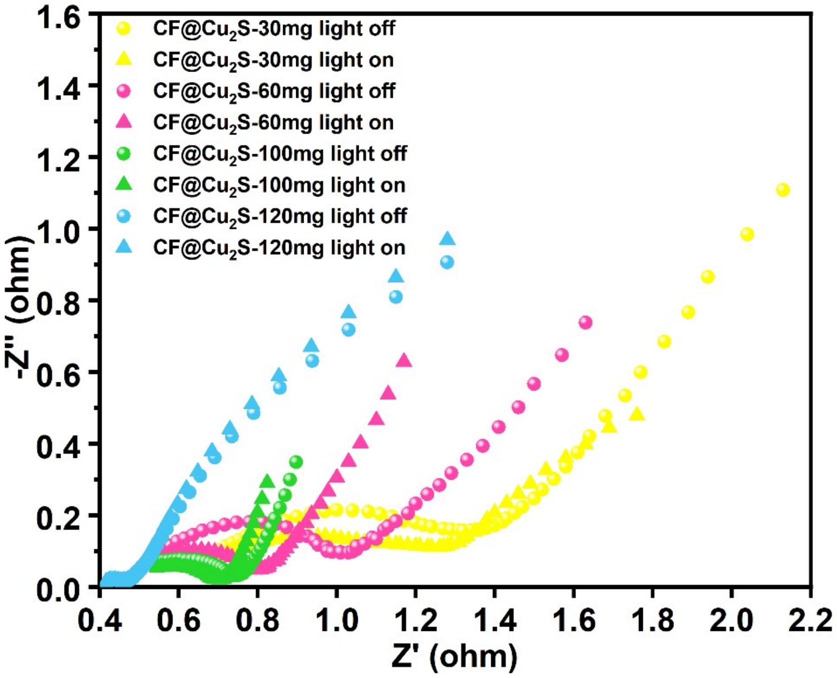

Fig. S3. EIS plots of CF@Cu_2S electrodes.

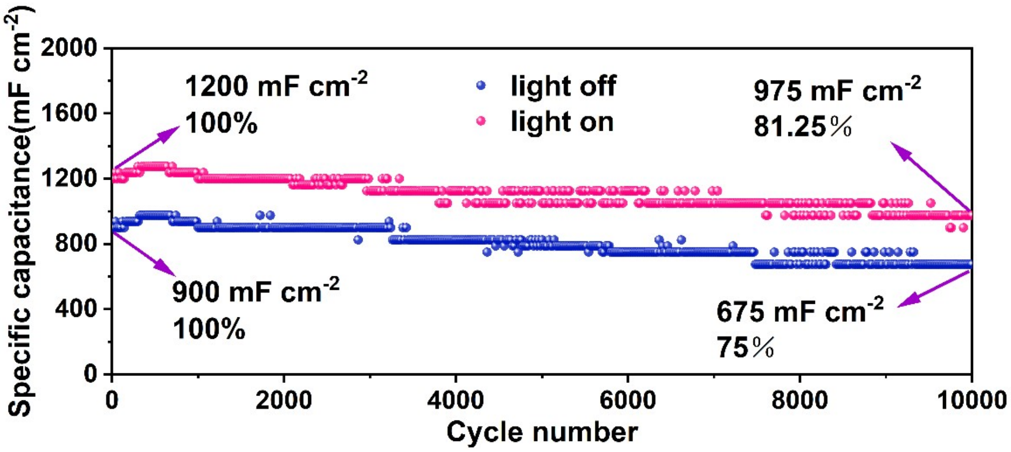

Fig. S4. Cycling performances under light off and on conditions of the CF@Cu_2S-100 sample at a current density of 30 mA cm^{-2} using a light source with a single wavelength of 365 nm.

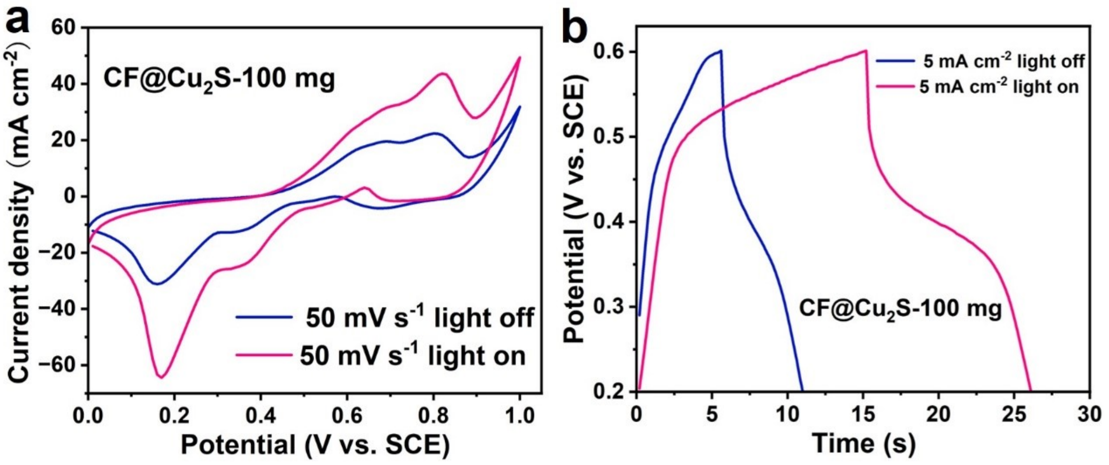

Fig. S5. (a) CV and (b) GCCD curves obtained under light on and off conditions, respectively, of the CF@Cu_2S-100 sample.

Table S1. A comparison of the electrochemical performance of CF@Cu_2S-100 with various PSC composites has been previously reported.

PSC devices	Capacitance (dark)	Capacitance (Light)	References
CL@SnS/NF	15.7 mF cm^{-2}	21.0 mF cm^{-2}	[1]
CoCN-0.55	943.8 mF cm^{-2}	1088.0 mF cm^{-2}	[2]
Cu_2O/Fe_2O_3	507 F g^{-1}	595 F g^{-1}	[3]
BVO-V_2O_5@TiNT	100 mF cm^{-2}	288 mF cm^{-2}	[4]
$NiCo_2S_4$@Cu_2O@CF	733.3 mF cm^{-2}	1156.7 mF cm^{-2}	[5]
**CF@Cu_2S-100**	**918 mF cm^{-2}**	**1458 mF cm^{-2}**	**This work**

References:

1. Li, T., et al., *Flake-like SnS anchored on the carbon layer for supercapacitor electrode with photo-responsive capacitance enhancement.* Journal of Alloys and Compounds, 2024. **976**: p. 173377.
2. Bai, L., et al., *Photocatalysis-assisted Co_3O_4/g-C_3N_4 p–n junction all-solid-state supercapacitors: a bridge between energy storage and photocatalysis.* Advanced science, 2020. **7**(22): p. 2001939.
3. Lv, X., et al., *Construction of light-sensitive Cu_2O/Fe_2O_3 heterostructures to*

promote photocatalytic CO_2 *reduction and photo-assisted charge storage.* Sustainable Energy & Fuels, 2024. **8**(21): p. 4992-5000.
4. Renani, A.S., et al., *New photoelectrodes based on bismuth vanadate-*V_2O_5*@ TiNT for photo-rechargeable supercapacitors.* Journal of Energy Storage, 2023. **62**: p. 106866.
5. Arif, M., et al., *Construction of three-dimensional* $NiCo_2S_4$*@*Cu_2O *nanowires with a high surface photovoltage to promote the energy efficiency of photo-assisted supercapacitors.* Journal of Colloid and Interface Science, 2025: p. 138067.
